# The Feasibility of a Novel School Peer-Led Mentoring Model to Improve the Physical Activity Levels and Sedentary Time of Adolescent Girls: The Girls Peer Activity (G-PACT) Project

**DOI:** 10.3390/children5060067

**Published:** 2018-05-31

**Authors:** Michael B. Owen, Charlotte Kerner, Sarah L. Taylor, Robert J. Noonan, Lisa Newson, Maria-Christina Kosteli, Whitney B. Curry, Stuart J. Fairclough

**Affiliations:** 1 Physical Activity and Health Research Group, Department of Sport and Physical Activity, Edge Hill University, St. Helens Road, Ormskirk, Lancashire L39 4QP, UK; Charlotte.Kerner@brunel.ac.uk (C.K.); Sarah.Taylor11@go.edgehill.ac.uk (S.L.T.); Robert.Noonan@edgehill.ac.uk (R.J.N.); Maria-christina.Kosteli@edgehill.ac.uk (M.-C.K.); Stuart.Fairclough@edgehill.ac.uk (S.J.F.); 2 Department of Life Sciences, Brunel University, London UB8 3PH, UK; 3 Natural Sciences and Psychology, Research Centre for Brain and Behaviour, Liverpool John Moores University, Liverpool L3 5AF, UK; L.M.Newson@ljmu.ac.uk; 4 Wellbeing and Public Health, Cornwall Council, Truro TR1 3AY, UK; Whitney.Curry@cornwall.gov.uk; 5 Department of Physical Education and Sport Sciences, University of Limerick, Limerick V94 T9PX, Ireland

**Keywords:** adolescents, girls, school, physical activity, sedentary time, intervention, peer-led, mentor, leader, accelerometry

## Abstract

Regular physical activity (PA) is associated with numerous physical and psychological health benefits. Adolescents, specifically girls, are at risk of physical inactivity. To date, there is limited research on PA interventions involving peers, which could encourage more adolescent girls to engage in PA. The investigation aimed to evaluate the feasibility of a novel school three-tier peer-led mentoring model designed to improve PA levels and reduce sedentary time (ST) of adolescent girls. Two-hundred and forty-nine Year 9 adolescent girls (13–15 years old) from three UK secondary schools were invited to participate in a peer-led mentoring intervention (Girls Peer Activity (G-PACT) project). The peer-led mentoring model was delivered in all three schools. Two of the schools received an additional after-school PA component. PA and ST were assessed through wrist-worn accelerometry. Girls who received an exercise class after-school component significantly increased their whole day moderate-to-vigorous PA (MVPA) (3.2 min, *p* = 0.009, *d* = 0.33). Girls who received no after-school component significantly decreased their MVPA (3.5 min, *p* = 0.016, *d* = 0.36) and increased their ST (17.2 min, *p* = 0.006, *d* = 0.43). The G-PACT intervention demonstrated feasibility of recruitment and data collection procedures for adolescent girls. The peer-led mentoring model shows promise for impacting girls’ MVPA levels when combined with an after-school club PA opportunity.

## 1. Introduction

Regular physical activity (PA) is associated with numerous health benefits for children and young people aged 5–18 years [1,2]. These include reduced body fat and the promotion of healthy weight, improved cardiometabolic and bone health, and enhanced psychological wellbeing [2,3]. Adolescents are particularly at risk of physical inactivity [4]. During adolescence biological and physiological changes occur, social priorities develop, and academic demands increase [5]. Globally, 80% of 13–15-year-olds do not engage in 60 min of moderate-to-vigorous PA (MVPA) per day, with girls being less active than boys [4,6,7]. For higher-risk adolescents (e.g., girls, overweight or obese) even modest amounts of PA can have health benefits [2], while replacing sedentary time (ST) with MVPA can have positive effects on body composition [8].

Previous school interventions have shown promise in slowing the decline in PA for this population [9,10,11]. Interventions underpinned with theory and that include multiple components, are the most promising approaches in the school setting [12,13]. Interventions promoting PA in the school environment delivered by older mentors or role models have been suggested as more appealing to adolescents than interventions delivered by teachers or researchers [9]. Adolescent health behaviours such as nutrition [14], smoking cessation [15], and sexual health [16] have been improved using cross-age mentorship interventions previously. However, cross-age mentorship has been understudied in PA research with young people [17,18].

Peer support, enjoyment, and perceived competence are significant influences on before-, during-, and after-school PA [19]. Adolescents with active friends are more likely to be physically active and spend less time engaging in screen-based behaviours [20]. It has been suggested that interventions aiming to increase MVPA in children and young people should be designed to include the recruitment of friends to increase enjoyment of MVPA [20]. One strategy that is relatively underused and consequently understudied in school PA interventions is the use of peer-led approaches [9,21,22,23]. Peer-led, peer leadership, and peer-assisted learning are terms that are frequently used interchangeably. The commonality is that each strategy is underpinned by a learning process whereby friends learn from and with others [24]. Peer-led involves similar aged peers [24], interacting with and motivating their classmates to initiate, continue, and sustain positive behaviour [15,25]. Importantly, not all peers are friends, thus, leadership selection is important to ensure peer-led interventions target a range of friendship groups. Previous interventions using the peer-led model to increase PA have shown the potential to increase girls’ MVPA [9,25]. Peer-led learning in combination with cross-age mentoring could be of benefit to adolescent girls. This innovative approach could provide another option to girls who may not be attracted to the sometimes competitive, rigorous, and potentially uncomfortable nature of traditional school-based PA [24].

The purpose of this investigation was to evaluate the feasibility of a novel school peer-led mentoring model designed to improve PA levels and reduce ST of adolescent girls (ages 13–15 years). This study aimed to (1) assess the feasibility of recruiting and retaining adolescent girls to a school peer-led mentoring intervention, (2) examine the feasibility of collecting accelerometer data to examine the PA levels and ST of adolescent girls, and (3) assess if a peer-led mentoring model can impact adolescent girls’ PA levels and ST.

## 2. Materials and Methods

### 2.1. Design

The Girls Peer Activity (G-PACT) Project was a three-arm, parallel group, non-randomised feasibility trial. Schools were allocated to each trial arm based on their ability and resources to implement the proposed intervention. The reporting of this study followed the CONSORT extension guidelines for feasibility and pilot trials [26]. Trial registration number: ISRCTN51511240.

### 2.2. Participants

Two-hundred and forty-nine Year 9 adolescent girls (13–15 years old) from three mixed-sex secondary schools situated in West Lancashire, north-west England, were invited to participate in the G-PACT project. The three secondary schools were located in areas with similar socioeconomic characteristics, based on the UK Indices of Multiple Deprivation (IMD) deciles (UK decile 6 or 7; [27,28]). The IMD is a UK Government measure comprising seven areas of deprivation, including income, employment, health, education, housing, environment, and crime. Year 9 girls (13–15 years old) were invited to participate in the project, and all data were collected at their respective schools.

### 2.3. Recruitment

A purposeful sampling strategy was used to recruit schools [29]. Firstly, emails were sent to Head of Physical Education (PE) departments at local schools inviting them to attend an initial meeting. The purpose of the meeting was to obtain input from the teachers regarding the intervention design, implementation, and evaluation. Prior to this study, one of the schools had previously participated in phase one of this project, an initial exploration study, which assessed girls’ PA levels and explored girls’ experiences and perceptions of school-based PA. With input from the research team, feedback from the PE teachers, and phase one data, a new 7-week peer-led school PA intervention was developed. Three schools that attended the original meetings, including the school used in phase one, were recruited to participate in the current intervention. The PE teachers in each respective school used convenience sampling during PE classes to recruit Year 9 girls to participate in the intervention. All girls were informed about data collection measures involved in the project and the additional intervention component (after-school PA opportunity) relating to their school.

After discussions with PE teachers, regarding suitability for the peer leadership role, 15 to 16 girls were recruited from PE cohorts to become Leaders in each school. The selection of the Leaders was initiated by the PE teachers based on a set of desirable role criteria (leadership abilities, communication skills, potential role model, confidence and social influence) as assessed by the teachers’ experiences with the girls. It was made clear to the teachers that the Leaders did not have to be “sporty” or physically active, as the intervention aimed to engage girls across all activity levels. This method was selected as it was presumed that the teachers would be best suited to identify girls meeting the criteria in their individual environments

#### Consent

Previous studies, [30,31] including school PA studies, [9,23,32,33] have utilised a passive consent (i.e., opt out) approach, rather than an active consent approach, with the former [30] being found to significantly increase participation (82% return for passive consent compared to 33% for letters direct to parents). Passive consent has previously been successful on a large-scale school PA peer-led approach in the UK [9]. The passive parents/carers consent method has been found to reduce sampling bias and be an ethical and appropriate way of informing parents/carers of “low-risk” prevention research [33]. During phase one of the G-PACT project, there was a low rate (26%) of returned informed active consent. Thus, the current study incorporated a passive consent approach, which had the full support of all participating schools. Ethical approval was granted from the Faculty of Arts and Sciences Research Ethics Committee at Edge Hill University (SPA-REC-2016-340).

### 2.4. Description of Intervention

**Figure 1:**
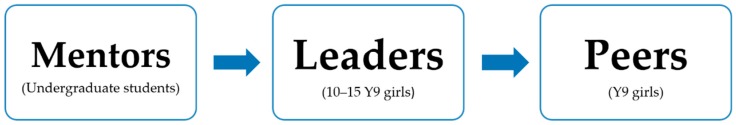
The intended direction of knowledge transfer from Mentors to Leaders to Peers.

The intervention incorporated a peer-led mentoring model based on Social Cognitive Theory (SCT) [34,35,36] and Self-determination Theory (SDT) [37]. This peer-led approach has been used previously in PA interventions with older children acting as Mentors [9,23]. The current study however, employed a novel approach by utilising a three-tier peer-led mentoring design. The intervention was developed with input from key stakeholders (PE teachers and adolescent girls) and G-PACT phase one data. The intervention incorporated a 7-week peer-led mentoring programme with an educational component that was consistent across all schools. As seen in Figure 1 below, the intervention had a three-tier design as follows: Mentors (undergraduate students), Leaders (Year 9 (Y9) girls selected by teachers), and Peers (whole Year 9 cohort). It was intended that these older Mentors would be role models as well as Mentors to the Leaders. The Mentors transfer their PA knowledge and leadership guidance to Leaders, who disseminate this knowledge to their Peers.

#### 2.4.1. Mentors

The Mentors were six final year undergraduate students (*n* = 6) studying Physical Education and School Sport Bachelor degrees at Edge Hill University. As part of their degree programme, Mentors had successfully completed a PA and health module, which included teaching on school-based interventions. The Mentors were purposely female, as it was anticipated this would help them build a greater rapport with the adolescent girls, and potentially be considered role models [34,35,36]. Each school had three Mentors supporting their Leaders, with some Mentors working in multiple schools. To ensure intervention fidelity and build rapport, the same three Mentors were present at their designated schools for all sessions.

#### 2.4.2. Educational Leadership Sessions

The Mentors delivered a series of educational leadership sessions for the Leaders which incorporated information on PA, health, motivation, barriers to PA, ideas to increase PA, ideas how to encourage Peers to be more physically active, and social support for their role (Table 1).

The educational sessions were theoretical (SDT and SCT) in their design, and applied multiple behaviour change techniques during delivery (e.g., goal setting, reviewing behaviour goals, social support, and problem solving [38]) (Supplementary Material. 1). The sessions were designed to increase levels of self-determination through the provision of opportunities to support girls’ three basic psychological needs of autonomy, competency, and relatedness [39]. The sessions also aimed to develop the Leaders’ self-efficacy to be physically active themselves, and to support their Peers to engage in more PA [35,36]. These sessions were all designed by the lead author, who used SDT and SCT to structure the session content and delivery approaches.

The educational sessions were solely delivered by the Mentors who received extensive training and ongoing supervision from the lead author. This training included information on different delivery methods, content knowledge, and theoretical underpinning. These educational sessions were designed to last no more than one hour in duration. The Mentors received a weekly checklist of content and tasks for each session, which they would complete after their session with the Leaders. This checklist was used to ensure continuity and consistency across the three intervention schools. As part of the checklist, Mentors were given the opportunity to discuss how the sessions were received by their Leaders. The Mentors met with the lead researcher on a weekly basis to feedback on the sessions, and to discuss the checklist. Mentors were provided with the opportunity to suggest additions or new delivery methods to best engage their leaders and to keep them engaged. During these feedback sessions, the content for the following week was also discussed along with how best to deliver the sessions. This method allowed the lead researcher to maintain consistency in delivery and content coverage of the intervention across the three schools.

This mentoring approach has been found to be appealing to adolescents, and has shown promise in influencing PA levels [9,40]. During the first session, the leaders were informed of their roles in the project, and discussed with mentors the best way to fulfil their roles and responsibilities within their respective schools. Through informal discussions, the Leaders were encouraged to disseminate the information they had learnt through their educational sessions to their friends and Peers. The Leaders were also asked to help to design information leaflets and posters to encourage more PA, including advertising the new after-school PA opportunities where appropriate. This peer-led approach, used as social influence through friends and peers, is crucial for adolescents to attain the best health behaviours in the transition into adulthood [5].

#### 2.4.3. Physical Activity Components

**Figure 2:**
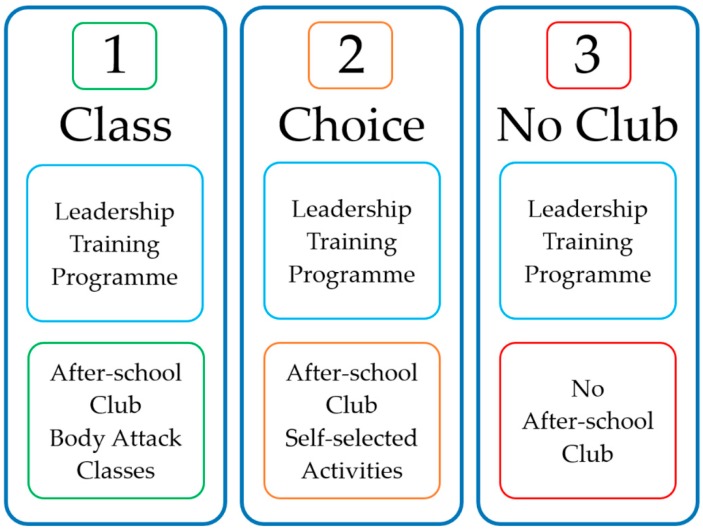
The educational information transfer and additional PA option for three different variations of the intervention (three separate schools).

In addition to the mentoring, educational sessions, peer-support, and information sharing that were consistent across all schools, there were three different PA session variations of the intervention (Figure 2). In conjunction with the education sessions, school one (Class) received weekly structured, class-based Les Mills *Body Attack* (https://www.lesmills.com/uk/workouts/group-fitness/bodyattack/) PA sessions delivered by trained and certificated instructors (relevant qualified undergraduate students). School two (Choice) received the option to choose what type of PA session they wanted to be part of their intervention. These sessions were designed with input from both Leaders and their Peers (multi-sports, dance, circuit training etc.). For school one and two, these PA sessions (approx. one hour in duration) ran weekly after school, from week 2 through to week 7. These after-school PA sessions had a maximum capacity of 30 girls due to space and resources restrictions. Finally, school three (No Club) did not receive an after-school PA component until after cessation of the intervention. The Leaders in the No Club school were asked to help develop this new after-school programme as part of their leadership role.

#### 2.4.4. Intervention Timeline

The intervention was delivered for 7 weeks. During week 1, after the Leaders were selected, they were invited by school to separately visit the university campus for a half-day workshop, delivered by their Mentors, introducing them to the project and their role within it. This session detailed the overall aim of the intervention, which was to increase PA levels of Year 9 adolescent girls in their respective schools, and how it was the Leaders’ role to encourage their friends to be more physically active over the next 7 weeks and beyond. This session was the same across all three schools. The remaining 6 weeks of the intervention were delivered on a weekly basis at the Leaders’ school. These sessions occurred at lunchtime or after school, depending on the facilities available in each school. Thus, Leaders from all three schools attended weekly leadership sessions (week 1–7) delivered by their Mentors, and the Leaders and Peers from school one and two had the option to attend the additional after-school PA sessions (week 2–7) which occurred on a different day of the week to the leadership sessions.

### 2.5. Measures

The impact of the intervention on adolescent girls’ PA levels and ST was assessed through 7-day wrist-worn accelerometry at baseline (week 0) and post-intervention (week 8). Supplementary self-reported measures of PA enjoyment, self-efficacy, wellbeing, and peer social support were taken at baseline and post-intervention. These measures were used to assess change in psychological states due to the intervention.

#### 2.5.1. Anthropometrics

Girls’ stature, weight, and waist circumference were measured using standardised procedures [41]. Stature was assessed to the nearest 0.1 cm using a portable stadiometer (Leicester Height Measure, Seca, Birmingham, UK). Body mass was assessed to the nearest 0.1 kg (761 scales, Seca). Body mass index (BMI) and weight status was calculated from stature and weight measurements as a proxy measure of adiposity [42]. BMI z-scores were calculated, and UK age and sex specific BMI cut points applied to categorise girls as underweight, normal weight, or overweight/obese [42,43]. Predicted age at peak height velocity (APHV) was used as a proxy measure of biological maturation using gender-specific equations [44]. Waist circumference was measured using an anthropometric tape to the nearest 0.1 cm. A measure of central adiposity was calculated using the waist circumference-to-height ratio (WHtR) [45], with 0.5 set as the global boundary for cardiometabolic risk [46]. All anthropometric measurements were conducted in a private area (not overlooked) in schools, by trained female research assistants under the supervision of the lead researcher. Some research assistants also had a role in the intervention as Mentors.

#### 2.5.2. Socioeconomic Status

Neighbourhood-level socioeconomic status was calculated from reported home postcodes using the 2015 IMD calculator [27]. The IMD is a UK government-produced measure composed of seven areas of deprivation (income, employment, health, education, housing, environment, and crime). IMD rank scores were matched to their corresponding IMD deciles, where decile 1 represents the most deprived 10% of areas nationally, and decile 10 the least deprived 10% of areas.

#### 2.5.3. Recruitment (Aim 1)

Counts and proportions of the number of girls in the schools providing passive parental consent were used to address Aim 1. This approach has been used previously in feasibility studies [47].

#### 2.5.4. Physical Activity Outcomes (Aims 2 and 3)

In order to assess the feasibility of collecting accelerometer data (Aim 2) and to assess the impact of a peer-led mentoring model on girls’ PA levels (Aim 3) all participants were asked to wear a wrist worn tri-axial accelerometer (ActiGraph GT9X, theActiGraph.com, FL, USA) to provide objective estimates of PA. These wrist-worn devices have been found to be a valid measure of PA [48,49,50]. With children and young people, wrist-worn devices reduce missing data and improve wear time compliance [49,50], which increases the accuracy of PA estimates. Girls were instructed to wear the devices on their non-dominate wrist for seven consecutive days. The accelerometers were positioned on top of the non-dominant wrist, proximal to the ulnar styloid process, so that the vertical axis of the ActiGraph was parallel to the longitudinal axis of the lower arm [51]. The instruments were worn over a period of seven days, to provide a reliable estimate of usual PA behaviour on weekend days and weekdays [52]. An information sheet regarding device use was given at both measurement points. Girls were instructed to wear the devices all the time (24 h·day^−1^) except when engaging in water-based activities, such as swimming or bathing.

Data collection took place during the school term from January to March 2017, therefore, data were representative of usual winter/spring free-living activities. Accelerometers were synchronised with Greenwich Mean Time (GMT), and initialised to record raw accelerations at a frequency of 100 Hz. After seven days of wear, the accelerometers were downloaded in ActiLife (v.6.11.8, ActiGraph) and saved in raw format (GT3X files). These raw files were then converted into CSV format to facilitate raw data processing in R (https://cran.r-project.org) using the GGIR package (v.1.5–17) [53]. The GGIR package converted raw tri-axial accelerometer signals [54] into one omnidirectional measure of acceleration termed “Signal Vector Magnitude” (SVM). SVM was calculated from raw accelerations from the three axes minus 1 *g* which represents the value of gravity (i.e., SVM = √(x^2^ + y^2^ + z^2^) − 1), after which negative values were rounded to zero. This metric has previously been referred to as the Euclidean norm minus one (ENMO) [53,54]. ENMO values were further reduced by calculating the average per 1 s epoch (expressed in mg) over the seven monitored days [49,55].

Accelerometer wear time periods for raw data were estimated on the basis of the standard deviation and value range of each axis, calculated for 60 min moving windows with 15 min increments [53]. This approach has been applied previously in ActiGraph studies involving youths [49,55,56,57]. A time window was classified as non-wear time if, for at least two out of the three axes, the standard deviation was less than 13.0 mg, or if the value range was less than 50 mg [58]. Accelerometer wear time inclusion criteria were at least 10 h of wear for a minimum of three weekdays. These wear time inclusion criteria have previously been used with school PA interventions exploring the whole day and school day PA levels [47,59,60,61], and is sufficient to produce reliable estimates of PA [62]. Published ENMO prediction equations were used to identify cut-points for classifying MVPA (3 METs (child-adjusted) = 201 mg) [63]. However, there is no consensus as to the most appropriate ENMO ST cut-points for adolescents [64]. Thus, we applied the Hildebrand et al. [63] regression equations using 1.5 METs (child-adjusted), which resulted in a value of 50 mg for the ST cut-point.

#### 2.5.5. Psychological Outcomes

A paper-based survey was administered to assess four psychological outcomes. The survey consisted of four components: 7-item PA enjoyment scale [65,66], 7-item wellbeing scale [67], 10-item social support scale [68], and 8-item self-efficacy scale [69,70]. The questionnaires have been validated and previously used with adolescents [70,71,72,73]. The surveys were completed at the start of the girls’ PE lesson under the guidance of a class teacher and at least two research assistants.

### 2.6. Data Analysis

Individual and school level descriptive statistics (mean and SD) were calculated for all measured variables including, the proportion of adolescent girls meeting the recommended daily 60 min of MVPA guidelines [1]. Recruitment figures and accelerometer data provision were calculated by school level. The primary outcome variables were ST and MVPA. Psychological outcomes were secondary outcomes; Cronbach’s alpha was used to test the questionnaires internal reliability. Raw data were checked for normality through visual (histograms, box-plots, Q–Q-plots) and parametric (K–S-test) assessments. Once normal distributions were confirmed, repeated measures ANCOVA were conducted to compare the three schools from baseline to post-intervention (post-INT) for primary and secondary outcomes. Three time windows: whole day (7 a.m.–11 p.m.), school day (9 a.m.–3.15 p.m.) and after-school (3.15 p.m.–4.45 p.m.) were analysed to examine differences across the three schools for ST and MVPA. In each ANCOVA, adjustments were made for baseline BMI, PA enjoyment, wear time, and ST or MVPA, respectively. If significant *time × school* interactions were observed, school-specific pairwise comparisons were made to investigate the differences over time. Subgroup analyses were conducted to investigate the differences for Leaders only and Peers only, using the same ANCOVA procedures detailed above. Effect sizes were calculated using Cohens *d* for larger samples (k > 20) (all girls and Peers only), and Hedge’s *g* for smaller sample sizes (k < 20) (Leaders) which provides a correction based on sample size. All analyses were conducted using IBM SPSS Statistics v.23 (IBM, Armonk, NY, USA) and statistical significance was set at 0.05.

## 3. Results

### 3.1. Recruitment and Data Provision

The participant recruitment and baseline data provision rates are shown by school in Table 2. The passive consent approach achieved a 94% recruitment rate with only 15 (6%) girls opting out of the whole project. Valid baseline accelerometer data were collected from 206 (88%) of the 234 consenting participants. The provision of valid accelerometer data was greater at baseline than post-INT (66%). This resulted in a reasonable baseline to post-INT attrition rate, with 76% completing all measures.

### 3.2. Descriptive Information

Descriptive and anthropometric characteristics of the participants are displayed in Table 3. There were no significant between group differences. The weight status calculation indicated that at baseline, 28% of the girls were overweight or obese, 67% of the girls were of a healthy weight and 5% were underweight. Only 2% of all girls across the three schools met the recommend daily MVPA guidelines at baseline.

### 3.3. Whole Day (7 a.m.–11 p.m.) PA Data

There was a main effect for *time* (*p* < 0.001) and a *time × school* effect (*p* = 0.026) for girls’ whole day ST (Table 4). Paired *t*-tests revealed a significant increase in girls’ whole day ST (17.2 min, *p* = 0.006, *d* = 0.43) for No Club school.

There was no main effect for *time* (*p* > 0.05) but, there was a *time × school* effect (*p* = 0.004) for MVPA (Table 4). The girls in the *Class* school significantly increased their whole day MVPA by just over 3 min (3.2 min, *p* = 0.009, *d* = 0.33). Whereas, the girls in the *No Club* school significantly decreased their MVPA levels by just over 3 min from baseline to post-INT (−3.5 min, *p* = 0.016, *d* = 0.36).

### 3.4. School Day (9 a.m.–3.15 p.m.) PA Data

There was a main effect for *time* (*p* = 0.004) and a *time × school* effect (*p* < 0.001) for school day ST. The girls in the No Club school significantly increased their school day ST by 14.0 min (*p* < 0.001, *d* = 0.90) (Table 5). There was no main effect for *time* (*p* > 0.05) but there was a *time × school* effect (*p* < 0.001) for MVPA. The girls in Class school significantly increased their school day MVPA levels by 1.2 min (*p* = 0.004, *d* = 0.37). Whereas, the girls in No Club school significantly decreased their MVPA levels by 2.7 min from baseline to post-INT (*p* < 0.001, *d* = 0.79).

### 3.5. After-School Club Period (3.15 p.m.–4.45 p.m.)

There was a significant main effect for *time* in after-school club period ST (*p* = 0.006) but not for MVPA (*p* > 0.05). However, between 3.15 p.m. and 4.45 p.m. there was no *time × school* effect (*p* > 0.05) for ST or MVPA (Supplementary Material. 2).

### 3.6. Subgroup Analyses

#### 3.6.1. Leaders

There was no main effect for *time* (*p* > 0.05) but, there was a *time × school* effect (*p* = 0.012) for Leaders’ whole day MVPA levels. Between 7 a.m. and 11 p.m., the Leaders from *No Club* school significantly reduced their MVPA levels (−9.3 min, *p* = 0.002, *g* = 1.07). There was no change for *Class* or *Choice* Leaders. There was no main effect for *time* or between group effects (both *p* > 0.05) for Leaders school day ST or MVPA levels. During the after-school period, there was no main effect for *time* for Leaders’ ST and MVPA (both *p* > 0.05) but, there was a *time × school* effect for Leaders’ MVPA (*p* = 0.012) and ST (*p* = 0.021). The *No Club* school Leaders significantly increased their ST (8.4 min, *p* = 0.002, *g* = 1.05) and decreased their MVPA levels (−3.2 min, *p* < 0.001, *g* = 1.50).

#### 3.6.2. Peers

Between 7 a.m. and 11 p.m., there was a main effect for *time* for Peers’ ST (*p* < 0.001) but not for MVPA (*p* > 05). There was no *time × school* effect for Peers’ ST or MVPA (both *p* > 0.05). Between 9 a.m. and 3:15 p.m., there was a main effect for *time* for Peers’ ST (*p* < 0.001) but not for MVPA (*p* > 0.05). However, there was a *time × school* effect for Peers’ school day ST and MVPA (both *p* < 0.001). During the school day, Peers from *No Club* school significantly increased their ST (15.5 min, *p* < 0.001, *g* = 0.76) and decreased their MVPA levels (−2.9 min, *p* < 0.001, *g* = 0.64). During the after-school period, there was a main effect for *time* for Peers’ ST (*p* < 0.001) but not for MVPA (*p* > 0.05) and there was a *time × school* effect for Peers’ ST (*p* = 0.042). Peers from *Class* school significantly decreased their after-school ST (−2.3 min, *p* = 0.005, *g* = 0.33).

### 3.7. Psychological Outcomes

All four questionnaires had good internal consistency with Cronbach’s alpha values >0.80 (social support (0.89), PA enjoyment (0.92), wellbeing (0.83), and self-efficacy (0.85)). There were positive main effects for *time* for social support, wellbeing, self-efficacy, and PA enjoyment (all *p* < 0.001). After controlling for differences at baseline, there were no *time × school* effects (*p* > 0.05) for social support, wellbeing, or self-efficacy for all participants. However, there was a *time × school* effect for PA enjoyment (*p* = 0.034). Paired *t*-tests revealed a significant increase in PA enjoyment for girls in *Class* school (*p* = 0.009, *d* = 0.28) but not for girls in *Choice* or *No Club* schools.

## 4. Discussion

The purpose of this investigation was to evaluate the feasibility of a peer-led school PA intervention for adolescent girls. The results show that it was feasible to recruit and retain adolescent girls to the project (intervention attrition 24%). There was a significant intervention effect on girls’ whole day and school day MVPA levels for *Class* school, and a negative impact on whole day and school day MVPA and ST for *No Club* school. The *Choice* school showed no significant differences across the two main outcome variables, but trends were in a positive direction for girls’ whole day and school day MVPA levels.

The passive consent (opt-out) approach appears crucial to the feasibility of this recruitment process. The passive consent approach utilised returned a recruitment rate of 94% from the eligible girls in Year 9. This is higher than previous studies using similar methods with this population (78% and 77%) [9] and significantly higher than opt-in consent (23%) [74]. Critically, this approach did not require any active engagement from the parents/carers unless desired. Passive consent is also used in England’s National Child Measurement Programme, which involves primary school children (aged 4–5 and 10–11 years old) having their height and weight measured [75]. In the current study, adolescent girls and their parents/carers were provided with multiple opportunities to opt-out, including for each individual measure (height, weight, accelerometer etc.). For low-risk non-intrusive research [33], such as the current study, this approach allowed for greater recruitment of adolescent girls, who can be a difficult population to reach. For these reasons, this approach should be considered by others doing similar work in the future.

The uptake in the after-school club opportunities was relatively low, however, this is common during this time period with adolescent girls [76]. The after-school Bristol Girls Dance Project (BGDP) had a mean attendance of 13 girls per session (max = 32) [47,76] which was similar to the attendance observed in the current study (14 and 12 girls per session (max = 30) in each *Class* and *Choice* school, respectively), illustrating the difficulties recruiting and retaining adolescent girls specifically in after-school PA clubs. Low uptake in the after-school clubs could have been due to time conflicts after school for adolescent girls or the advertisement strategies used in school.

It was feasible to collect accelerometer data with adolescent girls. A 24% data attrition rate from baseline to post-INT is comparable to previous PA assessments with adolescent girls. There was a 11% and 20% (week 9 and week 20) attrition rate in the BGDP [47] and in the larger Go-Active intervention pilot trial, 45% attrition (week 8) [9]. Girls were easily retained through the school-based structure and passive nature of the intervention design. Other than the after-school club opportunities, the Peers had little direct active engagement in the intervention. The intervention was largely disseminated through the Leaders who were constantly supported by their Mentors. Despite high recruitment and retention, the number of girls providing valid accelerometer data was low. This was predominantly due to difficulties with device wear time compliance, and provision of valid baseline and post-INT data. Three weekdays of 10 h wear time is a stringent inclusion criterion, but is commonly used to provide an accurate reflection of habitual weekday PA [60,61]. To increase compliance to device wear, multiple reminders during the measurement period or incentives could have been provided [9], but lack of resources and funding prohibited this strategy.

The *Class* school which received the class-based Les Mills *Body Attack* (https://www.lesmills.com/uk/workouts/group-fitness/bodyattack/), in addition to the leadership training programme, saw the greatest improvements in the outcome variables, including PA enjoyment. Multicomponent interventions compared to single component have been found to be more effective in impacting girls’ PA levels [12]. This infers that this class-based approach combined with the peer-led approach may have contributed towards the positive impact. The *Body Attack* sessions combined movements including running, lunging, and jumping with body weight strength exercises, such as push-ups and squats. These classes are primarily designed for older adolescents or adults. This more mature form of fitness-based class, which was non-competitive and non-team based, could have appealed more to the current adolescent girls [77,78], and been more conducive to MVPA participation compared to other traditional approaches (e.g., netball or hockey) [79]. However, it must be noted that the Les Mills classes require certified instructors, which can be an additional cost. If this intervention was to be scaled-up, a more cost-effective fitness class alternative may have to be explored.

There were significant unfavourable changes in ST and MVPA for the *No Class* school that received the same leadership and educational training programme as the other schools, but received no additional after-school PA opportunity. This finding is consistent with previous systematic reviews of school-based PA interventions for adolescent girls, with single component interventions found to be less effective on impacting girls’ PA levels [12]. Across multiple time windows, MVPA decreased, and ST increased significantly. Having no additional after-school club to complement the leadership training and peer-led approach could have contributed towards the findings compared to the other schools in the intervention. However, the increases in ST were large (17 min whole day and 14 min school day), thus, the lack of an after-school component was unlikely to be the only contributing factor to the increase in ST. Similarly, the BGDP found no evidence that an after-school dance programme could increase girls (11–12 years old) PA [47,80], illustrating the difficulties of changing PA behaviours in adolescences. Nevertheless, not having a PA prompt (after-school PA opportunity) could have been a factor limiting the Leaders’ ability to motivate and support Peers. However, from the current results, it was difficult to infer the cause(s) of the findings. The peer-led approach by design makes it difficult to assess the implementation of the intervention [81,82]. There was no exact measure of volume, intensity, quality of delivery and participant responsiveness to the messages the Leaders passed on to their Peers, and how much coverage these intervention messages achieved across all Peers [82]. Qualitative accounts from the Leaders and Peers may illuminate some important contextual information to supplement and explain the quantitative findings.

The *Choice* school showed no significant changes from baseline to post-INT. This finding is similar to the Girls Active programme, which contained a peer-led element, and showed no change in MVPA at the 14 month follow-up [22]. However, the changes observed in the *Choice* school were in a positive direction which is consistent with the Go-Active peer-led intervention which also found a positive trend in MVPA levels [9]. With the well-established declines in adolescent girls’ PA levels [4,6], this finding was encouraging, as the intervention prevented the anticipated decline in PA. Providing choice and autonomy has been well established as a successful approach influencing youths’ PA enjoyment and engagement [37,77,83,84]. Enjoyment is a stable and consistent psychological construct, which predicts PA participation and adherence, thus, it is deemed to be crucial to health behaviour change in youth [85,86,87]. Therefore, with the provision of choice, it is possible that a longer-term intervention may have seen significant positive PA changes with this population. Compared to the more expensive Les Mills classes, this choice of after-school element would be relatively cheap to implement on a larger scale if the intervention was to be up-scaled.

The peer-led mentoring model with leadership training sessions was implemented across all schools, but the current findings infer varying impacts of the main intervention component. Due to the complex nature of the intervention design, intervention fidelity and implementation were difficult to assess. Nevertheless, checklists were used to provide structure and consistency across sessions. However, the Mentors’ interactions with Leaders, and Leaders’ subsequent interactions with Peers cannot be quantified, and could have influenced intervention fidelity and implementation. Peer-led approaches are novel in PA interventions with adolescents [18]. The current study aimed to assess the feasibility of this approach, in accordance with the Medical Research Council guidance for developing and evaluating complex interventions, it is expected that refinements and modifications are needed before further piloting the intervention [88,89]. The refinements or additions to the current project could include a greater emphasis on reducing ST during the school day, and the provision of more MVPA opportunities on multiple school days both during and after school. Comprehensive school-based approaches have been suggested as effective strategies to increase young peoples’ PA [90,91,92]. The G-PACT intervention was largely delivered though the PE departments of each individual school. By contrast, the Go-Active intervention adopted a wider school approach, incorporating intervention features into morning registration classes which showed initial success [9]. However, the Go-Active intervention was a mixed-sex intervention for adolescents, meaning this approach was easier to implement in a mixed-sex school.

### Strengths and Limitations

A major strength of this study was the novel peer-led mentoring model incorporating older Mentors. This peer-led mentoring model allowed for a three-tier knowledge transfer process and provided Leaders with support to aid their Peers’ PA behaviours. This mentoring approach could have relevance to other health related disciplines, such as positive lifestyle choices, including diet and nutrition. However, it is unclear if this collaborative approach between schools and a university would be sustainable over a longer period, due to university students’ time commitments and career progression. That said, once the Leaders have received extensive training and mentoring, less frequent meetings with the Mentors could be incorporated into the intervention design.

Another strength was the detailed recruitment and accelerometer data provision processes in a moderately sized feasibility trial, which can inform the design of future interventions. Additionally, the study achieved a high recruitment rate using the passive consent method with adolescent girls in the school setting. However, due to the recent General Data Protection Regulation (GDPR) changes (the GDPR is Europe-wide), active opt-in processes will be needed in future research with children and young people (<16 years old). This has implications for future school-based research, as active opt-in leads may reduce recruitment rates and result in less representative samples compared to passive opt-out consent [31]. Furthermore, the use of objectively measured PA and raw data processing to assess the intervention effectiveness helped avoid the uncertainty of pre-processed data, such as counts, and the possibility that signal filtering methods alter study results [93,94]. This school-based, girls only intervention was underpinned by two behaviour change theories (SDT and SCT), as recommended to promote intervention effectiveness [12,13].

It is, however, important to recognize that the data reported here originate from a non-randomised design, so there was greater potential for bias within the study. Further, there was no control group used, therefore, it is difficult to distinguish the true effect of the intervention. However, the MRC complex interventions guidance advises that if impractical, randomisation and the use of a control group is not essential in feasibility designs [88]. A longer follow-up period than 7 weeks could have been used to assess the intervention effects, but school-term time constraints and data collection resources did not allow for this. Another limitation of the study was that there was also no specific measure of motivation for PA, which could have been useful to assess the change in motivation and potential future intentions to engage in PA. Due to the design of the intervention, it was difficult to distinguish which girls benefitted from the peer-led aspect of the intervention, and how much impact this had on their PA behaviours. Tracking social networks could be considered in future peer-led interventions to assess the links between friends, and how this may develop as a result of the intervention [95].

All three schools were mixed-sex, which could have negatively influenced the intervention implementation. Girls may have not felt comfortable discussing PA around their male counterparts. Only a small percentage of the girls attended the weekly after-school PA club, thus, it was difficult to distinguish how beneficial this component was to changes in PA habits. The lead researcher was mainly responsible for ensuring consistency in delivery and content of the leadership training programme across the three schools. This could be seen as a potential source of bias, however, standardised checklists were used with the Mentors to provide structure to the sessions and reduce potential bias. These session checklists included coverage of the weekly content and delivery methods to ensure consistency across the three schools. Future studies should include process evaluation to allow implementation fidelity to be assessed [89].

## 5. Conclusions

This feasibility study of the G-PACT intervention showed feasibility of recruitment and data collection procedures for adolescent girls. The peer-led mentoring model shows promise for impacting girls’ PA levels when combined with an after-school club PA opportunity. The class intervention resulted in the most favourable changes in MVPA and ST. Moreover, the school that did not receive the after-school club alongside the peer-led mentoring model showed reductions in MVPA and increases in ST across the intervention duration, which suggests that this was an important component of the project. The peer-led mentoring model requires further investigation, including qualitative work, which could contextualise the quantitative results reported above. The peer-led mentoring model provides a novel method to target the PA behaviours of adolescent girls and their peers.

## Figures and Tables

**Table 1:** The 7-week leadership educational training programme for all three schools.

Week	Location	Activity
1	University	Introduction & Leadership & PA
2	School	PA & Motivation
3	School	PA & Goal setting
4	School	PA & How to increase activity
5	School	PA & How to support others
6	School	Support session
7	School	Support session

Notes: PA = physical activity

**Table 2:** Participant recruitment, opt-out rate, data provision, and mean weekly attendance for 6-week after-school club programme in the G-PACT project.

School	Number of Girls in Year Group	Opted Out (*n*, (%))	Provided Accelerometer Data (*n*, (%))		Weekly ASC Attendance (Mean, (%))
			Baseline *	Post-INT *	
1. Class	102	9 (8.8)	82 (88.2)	70 (75.3)	14 (47)
2. Choice	76	3 (3.9)	64 (87.7)	42 (57.5)	12 (40)
3. No Club	71	3 (4.2)	60 (88.2)	45 (66.2)	N/A
Total	249	15 (6.0)	206 (88.0)	157 (66.3)	N/A

Notes: ASC = after-school club; * Numbers represent provision of valid data (3 valid weekdays with minimum 10 h wear).

**Table 3:** Descriptive and anthropometric characteristics of participants by individual school and overall (mean (SD) or percentage).

	1. Class (*n* = 93)	2. Choice (*n* = 73)	3. No Club (*n* = 68)	All Girls (*n* = 234)
Age (y)	14.0 (0.3)	14.0 (0.3)	14.1 (0.3)	14.0 (0.3)
Stature (cm)	160.7 (5.8)	160.4 (5.7)	161.7 (9.5)	160.9 (6.9)
Body Mass (kg)	56.8 (10.2)	55.4 (8.8)	57.8 (12.1)	56.7 (10.5)
BMI (kg∙m^2^)	22.4 (5.9)	21.7 (3.2)	22.4 (6.6)	22.2 (5.6)
BMI z-Score	0.7 (1.0)	0.7 (0.9)	0.6 (1.4)	0.7 (1.1)
WHtR	0.46 (0.04)	0.44 (0.04)	0.46 (0.07)	0.45 (0.05)
Maturity Offset (y)	1.8 (0.4)	1.8 (0.4)	1.9 (0.6)	1.8 (0.5)
Weight Status (%)				
Underweight	2.2	5.5	9.2	5.2
Normal Weight	70.0	67.3	61.6	66.7
Overweight/Obese	27.8	27.3	29.2	28.1
IMD Score	6.6 (2.6)	5.7 (3.0)	6.8 (2.0)	6.4 (2.6)
MVPA (min·day^−1^)	23.3 (11.9)	26.4 (13.3)	32.2 (12.9)	26.9 (13.1)
MVPA Guidelines (%)	0	3.1	3.3	1.9

Notes: BMI = body mass index, WHtR = waist-to-height-ratio, Maturity Offset = predicated time from peak height velocity, IMD = indices of multiple deprivation, MVPA levels = mean moderate-to-vigorous PA (MVPA) level in minutes at baseline, MVPA Guidelines = % of girls meeting the recommended daily 60 min of MVPA at baseline.

**Table 4:** Whole day adjusted means (SE) for ST and MVPA at baseline, post-INT and mean difference.

School	Time	ST (Minutes)		Adjusted Difference in Means (95% CI)	MVPA (Minutes)		Adjusted Difference in Means (95% CI)
		Mean	SE		Mean	SE	
1. Class	Baseline	728.0	0.0	−1.6 (−10.0 to 7.5)	28.7	0.0	3.2 * (0.8 to 5.7)
	Post-INT	726.4	4.5		31.9	1.15	
2. Choice	Baseline	728.0	0.0	12.9 (2.0 to 20.3)	28.7	0.0	1.0 (−1.9 to 2.7)
	Post-INT	740.9	6.5		29.7	1.6	
3. No Club	Baseline	728.0	0.0	17.2 * (4.8 to 26.5)	28.7	0.0	−3.5 * (−5.8 to −0.5)
	Post-INT	745.8	5.7		25.2	1.4	

Notes: * *p* < 0.05, Mean Difference = change baseline to post-INT.

**Table 5:** School day adjusted means (SE) for ST and MVPA at baseline, post-INT and mean difference.

School	Time	ST (Minutes)		Adjusted Difference in Means (95% CI)	MVPA (Minutes)		Adjusted Difference in Means (95% CI)
		Mean	SE		Mean	SE	
1. Class	Baseline	281.3	0.0	−4.9 (−8.2 to −0.6)	10.6	0.0	1.2 * (0.5 to 2.5)
	Post-INT	276.4	2.0		11.8	0.6	
2. Choice	Baseline	281.3	0.0	−3.4 (−9.8 to 0.3)	10.6	0.0	1.9 (0.2 to 3.0)
	Post-INT	277.9	2.9		12.5	0.8	
3. No Club	Baseline	281.3	0.0	14.0 ** (9.5 to 18.8)	10.6	0.0	−2.7 ** (−4.6 to −2.1)
	Post-INT	295.3	2.6		7.9	0.7	

Notes: * *p* < 0.05, ** *p* < 0.001, Mean Difference = change baseline to post-INT.

## Supplementary Materials

The following are available online at http://www.mdpi.com/2227-9067/5/6/67/s1, S1: Educational leadership sessions theory map and session aims, S2: after-school period PA results table.
