# Supplementary material for: The Feasibility of a Novel School Peer-Led Mentoring Model to Improve the Physical Activity Levels and Sedentary Time of Adolescent Girls: The Girls Peer Activity (G-PACT) Project
Source: Children (Basel). 2018 May 31;5(6):67. doi: 10.3390/children5060067 (PMC6028908; doi:10.3390/children5060067)
Supplement: Supplementary file 1 [file children-05-00067-s001.pdf]

Supplementary Material

Title: The Feasibility of a Novel School Peer-led Mentoring Model to Improve the Physical Activity Levels and Sedentary Time of Adolescent Girls: The Girls' Peer Activity (G-PACT) Project

Michael B. Owen [1,*], Charlotte Kerner [1, 2], Sarah L. Taylor [1], Robert J. Noonan [1], Lisa Newson [3], Maria-Christina Kosteli [1], Whitney B. Curry [4], and Stuart J. Fairclough[1, 5]

1. Physical Activity and Health Research Group, Department of Sport and Physical Activity, Edge Hill University, St Helens Road, Ormskirk, Lancashire, L39 4QP, UK; Michael.Owen@edgehill.ac.uk (M.B.O.); Sarah.Taylor11@go.edgehill.ac.uk (S.L.T.); Robert.Noonan@edgehill.ac.uk (R.J.N.); Maria-Christina.Kosteli@edgehill.ac.uk (M.C.K.); Stuart.Fairclough@edgehill.ac.uk (S.J.F.)
2. Department of Life Sciences, Brunel University, London, UB8 3PH, UK; Charlotte.Kerner@brunel.ac.uk (C.K.)
3. Natural Sciences and Psychology, Research Centre for Brain and Behaviour, Liverpool John Moores University, Liverpool, L3 5AF, UK; L.M.Newson@ljmu.ac.uk (L.N.)
4. Wellbeing and Public Health, Cornwall Council, Truro, TR1 3AY, UK; whitney.curry@cornwall.gov.uk (W.B.C.)
5. Department of Physical Education and Sport Sciences, University of Limerick, Limerick, Ireland

* Correspondence: Michael.Owen@edgehill.ac.uk; Tel.: +44-016956-57344

S1: Educational leadership sessions theory map and session aims

**Session Focus**	**Session Overview / LO's**	**Behaviour Change Techniques**	**SCT / SDT Constructs**
Workshop Day Part 1; Introduction to programme and leadership Information	- What is the purpose of the project?<br>- Provide information about leadership and the role of peer leaders<br>- What do the students think about leadership?<br>- What are the traits of good leaders?	- Participation in *fun* workshop activities<br>- Prompt identification as role model/ position advocate<br>- General communication skills training	- Autonomy<br>- Competence<br>- Relatedness<br>- Intrinsic Motivation<br>- Role modelling<br>- Self-efficacy
Workshop Day Part 2; PA and health knowledge	- What is PA?<br>- What are the different type of PA?<br>- What physical activities do the students do?<br>- Importance of PA for good health, physical, psychological and social<br>- Communication task	- Provide information on consequences of behaviour in general<br>- Provide information on consequences of behaviour to the individual<br>- Provide normative information about others' behaviour<br>- Prompt identification as role model/ position advocate	- Autonomy<br>- Competence<br>- Outcome expectations<br>- Outcome expectations<br>- Outcome expectations<br>- Role modelling

School Workshop 1; Motivation	- What is motivation?<br>- How to motivate others?<br>- Review of first PA session	- Plan social support/ social change<br>- Barrier identification/Problem solving	- Autonomy<br>- Competence<br>- Social support
School Workshop 2; Goal setting	- What is goal setting?<br>- How to set effective goals?<br>- Develop goal setting skills / set targets to achieve	- Goal setting (behaviour)<br>- Prompt self-monitoring of behaviour including; setting goals, reviewing goals, specifying action plans and feedback on performance	- Autonomy<br>- Competence<br>- Social support<br>- Self-regulation
School Workshop 3; Barriers to PA and methods to increase PA	- Identify barriers to PA<br>- Methods to break down barriers<br>- More in-depth benefits of physical activity<br>- Problem solving task	- Barrier identification/Problem solving<br>- Provide information on where and when to perform the behaviour (Prompts cues)	- Self-efficacy<br>- Autonomy<br>- Competence<br>- Intrinsic Motivation
School Workshop 4; Social support and review of programme	- How to provide social support to friends<br>- How to encourage friends<br>- How to engage with peers	- Plan social support/ social change<br>- Barrier identification/Problem solving	- Social support<br>- Self-efficacy<br>- Autonomy<br>- Competence<br>- Relatedness

	- Review previously covered content for information booklet<br>- Problem solving task		
School Support Sessions 5 and 6; Leadership support and mentoring	- Revisit previously set goals<br>- Re-set goals for future weeks<br>- Provide the leaders support and advice on their role<br>- Discuss the new after school PA club an probe for feedback on the club<br>- Encourage peer leaders in their role	- Review behaviour goals (goal setting)<br>- Prompt self-monitoring of behaviour<br>- Prompt review of behavioural goals<br>- Barrier identification/Problem solving<br>- Provide information on where and when to perform the behaviour (Prompts cues)	- Autonomy<br>- Competence<br>- Relatedness<br>- Intrinsic Motivation<br>- Social support<br>- Self-regulation<br>- Self-efficacy

S2: After-school club period adjusted means (SE) for ST and MVPA at baseline, post-INT and mean difference

School	Time	ST (Minutes) Mean	ST (Minutes) SE	Adjusted difference in means (95% CI)	MVPA (Minutes) Mean	MVPA (Minutes) SE	Adjusted difference in means (95% CI)
**1.Class**	Baseline	62.9	0.0	-1.4 (-4.5 to -0.7)	5.1	0.0	0.0 (-0.1 to 1.2)
	Post-INT	61.5	1.1		5.1	0.4	
**2.Choice**	Baseline	62.9	0.0	2.7 (-0.3 to 5.8)	5.1	0.0	0.0 (-1.2 to 1.2)
	Post-INT	65.6	1.6		5.1	0.6	
**3.No Club**	Baseline	62.9	0.0	1.1 (-2.3 to 4.2)	5.1	0.0	-1.2 (-2.6 to -0.1)
	Post-INT	64.0	1.4		3.9	0.5	

Notes: Mean Difference = change baseline to post-INT
